# Outcasts and saboteurs: Intervention strategies to reduce the negative effects of social exclusion on team outcomes

**DOI:** 10.1371/journal.pone.0249851

**Published:** 2021-05-06

**Authors:** Andrew Reece, Evan W. Carr, Roy F. Baumeister, Gabriella Rosen Kellerman

**Affiliations:** 1 BetterUp, Inc., San Francisco, CA, United States of America; 2 School of Psychology, University of Queensland, St Lucia, Australia; University of Padova, ITALY

## Abstract

The experience of social exclusion in the workplace adversely impacts employees’ well-being, job satisfaction, and productivity, and no one quite knows what to do about it. In this report, we describe the development and testing of three ostracism interventions, designed to help people cope with the negative effects of being excluded by one’s team. Across five studies, participants were assigned to a virtual ball toss game where they were either included or excluded by their teammates. Afterwards, they were given a task where they could earn money for themselves, for their entire team, or for an unrelated group (charity). Excluded participants worked less hard for their teams (even when this meant sacrificing their own earnings). This sabotage effect was *specific*, meaning that excluded individuals worked less hard on behalf of their teams, but not when they worked for themselves or for charity. We devised three intervention strategies—perspective, mentorship, and empowerment—to combat the negative effects of ostracism on people’s willingness to work for their teams. These interventions were successful; each increased people’s persistence in a team-based reward task, and in some cases, even raised the outcomes of excluded teammates to levels observed in *included* teammates. The effectiveness of these interventions also replicated successfully, using preregistered hypotheses, methods, and analyses. These studies add novel insights to a variety of fields that have examined the consequences of social exclusion, including social psychology, organizational behavior, and management science.

## Introduction

The sting of social rejection is a uniquely painful and universally loathed experience. Urged on by this basic human experience, the last 50 years have seen countless articles written on social exclusion and ostracism across a variety of fields. Few of these articles seek applied solutions to reduce the harmful effects of ostracism, which many consider a growing epidemic, especially among younger generations [1]. The present investigation first established some of the antisocial tendencies that follow from social exclusion, and then tested three possible interventions to help people cope with the negative effects of ostracism.

Such troubles with social rejection and ostracism deeply affect people’s professional lives. More than 70% of professionals report experiencing social exclusion or isolation, which research suggests can be as psychologically harmful (if not more so) than being targeted by more overt antisocial behaviors [2]. Instances of social exclusion can lead to longer-term physical and emotional isolation in the workplace [3], which 40% of people report experiencing. This in turn has consequences for both employees and organizations, such as decreased loyalty, reduced commitment, and higher turnover [2]. These negative effects of workplace exclusion are especially concerning given that the excluding parties do not always recognize their behaviors as hurtful [4, 5]. Many organizations now recognize workplace exclusion as a major concern [6] and have devoted considerable resources to inclusion training initiatives [7], many of which fail due to a lack of empirically supported solutions.

A review of the existing literature on social exclusion reveals a sprawling landscape of terms, manipulations, theories, and frameworks [8–10]. At its most basic, the experience of *ostracism*—being excluded and ignored by individuals or groups [11]—arises within interpersonal relationships [12], occurs across a range of societal and institutional circumstances [13], and is often used as a tool for social influence [14, 15]. While the majority of social psychological research on ostracism took place after 1990 [16], earlier studies also documented the power and pervasiveness of the phenomenon. For instance, Schachter (1951) [17] noted “the consequences of deviation from a group standard,” when deviates did not conform to a common opinion. Even William James described the experience of exclusion in his writings on the social self back in 1890 [18]:

“No more fiendish punishment could be devised, were such a thing physically possible, than that one should be turned loose in society and remain absolutely unnoticed by all the members thereof. If no one turned round when we entered, answered when we spoke, or minded what we did, but if every person we met ‘cut us dead,’ and acted as if we were non-existing things, a kind of rage and impotent despair would ere long well up in us, from which the cruelest bodily tortures would be a relief” (p. 293–294).

Despite its long history of consideration by psychological and management science, to date no reliable means of relief from James’ “fiendish punishment” has yet been uncovered. In this paper, we seek to change that by focusing on two questions, with specific importance for the workplace: First, when team members are excluded, does this have negative consequences for the team? And second, once exclusion occurs, what interventions (if any) can be used to help people cope with the negative effects of ostracism?

### Responses to exclusion can be prosocial or antisocial

Generally, once someone has been excluded, their reaction falls into one of two categories [19]: *prosocial* responses, which are thoughts and behaviors that have positive social consequences and/or aim to improve interpersonal connections, or *antisocial* responses, which are thoughts and behaviors that have negative social consequences and/or amplify the division between the group and excluded individual. Several meta-analyses and theoretical papers have made strides towards outlining when and where these responses occur [8, 9, 11, 16, 20–22]. For example, *prosocial* responses come in many forms, often owing their character to the underlying needs that are being threatened by the act of exclusion. For example, excluded individuals may ingratiate themselves to the excluders [23, 24], increase their compliance [25], express liking for new groups [21], unconsciously mimic others’ behaviors [26], turn to alternative affiliation sources [27, 28], and increase ingratiatory and citizenship behaviors at work [29]. These reactions could be motivated by the desire to restore the thwarted psychological need to belong [30, 31].

On the other hand, *antisocial* responses to social exclusion can arise in the form of aggressive or vengeful acts [32–39], pursuit of risky or self-defeating behaviors [40–43], or becoming a disengaged “social loafer” [44–46]. Antisocial responses to rejection may serve as attempts to rebalance a thwarted psychological need for control over one’s environment [9, 37, 47, 48]. These reactions might be especially likely in scenarios that do not prompt a strong motivation for reinclusion in the group [9, 21].

A similar contrast between prosocial and antisocial responses to ostracism is also present in the organizational behavior literature. Social exclusion has been repeatedly associated with antisocial responses at work that hinder productivity and performance [21, 49–51], reduce employee citizenship behaviors [52], lower job satisfaction [20], and hamper personal and emotional well-being [53]. Conversely, studies also indicate that workplace ostracism sometimes increases employees’ prosocial behavior, likely as an attempt be re-accepted by one’s group [46, 54]. Once again, these opposing findings have prompted scholars to search for a reconciliation. Much like the broader ostracism literature, any reconciliation of these prosocial and antisocial tendencies requires a deeper understanding of mechanism. To this end, researchers have explored many possible reasons for employees’ response to ostracism, including the effects of ostracism on one’s self-esteem, one’s sense of obligation towards one fellow employees, or the extent to which ostracized individuals identify with their organizations [52, 55, 56]. More broadly, researchers have also tried to explain both prosocial and antisocial tendencies using a single framework; for example, from social dilemma perspective, the tendency of ostracized employees to engage in prosocial or antisocial behaviors might depend on whether a short-term prosocial response would allow them to achieve a longer-term goal [57].

In this report, we focus specifically on the antisocial effects of social exclusion. In other words, our primary aim is not to provide a theoretical reconciliation of these discrepant prosocial versus antisocial reactions. Instead, we simply acknowledge the existing empirical complexity—that of the different possible reactions to ostracism, as well as the complexity associated with elucidating why these different reactions occur. Here, we simply start from the premise that in a substantial number of cases, ostracism is associated with negative antisocial responses, both in general and in the workplace [49–51]. Our goal was to create an experimental setup that reliably induces these antisocial responses to ostracism, after which we aimed to provide empirical evidence for some possible intervention strategies that might work to mitigate these negative effects.

As such, our experimental paradigm used a popular psychological manipulation, called *Cyberball*, which has been shown to reliably induce social inclusion or exclusion [58], usually resulting in antisocial reactions. This experimental setup allowed us to address our main goal of demonstrating the negative effects of exclusion on people’s willingness to work for their teams, and then testing different interventions intended to quell those negative effects. In consideration of prior research on behavioral responses to exclusion (and specifically, to cyberball-induced exclusion), our first hypothesis was as follows:

H_1_: In our experiments, participants will display *antisocial* behavior in response to being excluded. Specifically, they will work less hard on behalf of their teams. This should occur even when these antisocial responses are self-defeating (e.g., causing the excluded individual to earn less money themselves).

An addendum to this first hypothesis involves the potential target of any antisocial response. Previous research [11, 19] has demonstrated that antisocial responses after exclusion can be *specific* (where the excluded individual selectively punishes members of the excluding group) or *general* (where negative responses are directed toward any source, once exclusion has occurred). In our experiments, once participants were excluded by their teams, they had the chance to earn additional rewards on behalf of their team (based on their willingness to persist on a simple task). This measure of task persistence is our main dependent variable, and in one of our experiments (Study 3), we manipulate whether excluded individuals are earning money for themselves, their team, or another uninvolved group (charity). This leads to our second hypothesis:

H_2_: Excluded participants will engage in *specific* antisocial responses (i.e., selectively punishing one’s teammates who perpetrated the exclusion), rather than general antisocial responses (i.e., punishing those who were not associated with the act of exclusion).

Our rationale for Hypothesis 2 follows from prior research in organizational behavior, game theory, developmental psychology, and neuroscience, which has demonstrated that people are acutely aware of fairness considerations [35, 59]. As a result, people tend to seek retribution for observed and experienced unfairness [32, 60], often by punishing the excluders [28, 61], even at a cost to themselves [39]. Studies on revenge and retributive justice have also shown that the type and intensity of antisocial responses can depend on factors like group values [62], hierarchical status [63], intentionality [62], and goals associated with retaliation [62, 64, 65], all of which can lead to workplace sabotage [66, 67]. Overall, because exclusion from their team is likely perceived as a highly non-cooperative act, we expect people to not only have antisocial responses to the exclusion (Hypothesis 1), but also that their reactions would be *specific* and targeted towards the teammates that perpetrated the exclusion in the first place (Hypothesis 2).

In sum, the primary goal of this investigation is not to adjudicate between or provide a full exploration of the underlying mechanisms of prosocial versus antisocial responses to ostracism. Rather our goal is to build on previous research demonstrating the ubiquity and importance of antisocial responses to ostracism, while providing possible intervention strategies for people to cope with ostracism once it has occurred. We provide a fuller description of the context, motivation, and details surrounding these interventions in the following sections.

### Targeted interventions may reduce the negative consequences of exclusion

If we are correct in Hypotheses 1–2, the effects of social exclusion will be antisocial and specific, leading excluded individuals to work less on behalf of their teams, even at a cost to themselves. Given the surge in ostracism research since the 1990s [16], there have been repeated calls to shift the focus to more applied work on evidence-based interventions to combat the negative effects of ostracism [68, 69]. Our main goal was to start to address this gap: After exclusion occurs, what interventions (if any) can be used to help people cope with the negative effects of ostracism?

While there has been some applied research on ostracism interventions, our work is distinct in that we offer strategies to cope with ostracism that has already occurred. As a comparison, Leiter and colleagues (2011) examined an intervention called CREW (Civility, Respect, and Engagement at Work) designed to reduce incivility and improve employee outcomes in a cohort of 1,173 healthcare workers. They found significant improvements on various indicators of social relationships, burnout, turnover intention, attitudes, and management trust, suggesting that such interventions can lead to positive changes in company culture. Note, however, that the CREW intervention is designed to proactively reduce *overall* levels of ostracism and incivility, rather than a more targeted approach to address ostracism as it occurs (as with our studies). Our interventions also have the added benefit of taking only a few minutes to implement (compared to CREW, which took place over the course of 6 months). Both angles are obviously important, but given that some level of workplace ostracism is inevitable [10, 11, 22, 62], especially since people often do not even recognize when they are excluding others [4, 5], we believe our interventions are both novel and impactful.

Other applied studies report promising results for the social and organizational benefits of ostracism interventions. For example, social belonging interventions in academic environments (e.g., reading about previous students’ hardships) have been shown to improve academic achievement in minority groups [70] and help integrate women into fields dominated by men [71]. There is also suggestive evidence that ostracism interventions might be helpful in team contexts. Another study showed that a simple acknowledgement after exclusion can improve need satisfaction [72], which is inextricably linked to the behaviors that follow exclusion [73, 74]. These articles led us to our next hypothesis, regarding the potential timing and effectiveness of exclusion interventions:

H_3_: After participants have been excluded, the negative effects on their persistence in a team-based reward task will be mitigated if they engage in an applied intervention.

Note that there are two key components to Hypothesis 3. One pertains to the expected effectiveness of the interventions; a full mitigation of exclusion-induced deficits to task persistence would imply that an intervention had improved *excluded* individuals’ task persistence to that of participants who were *included* by their group. While this is an admittedly high bar, the aforementioned intervention articles report impressive effects [70, 71], even if the intervention is relatively brief and minimal [75]. The second aspect pertains to the timing, in delivering an exclusion intervention immediately after the exclusionary event occurs but before a team-reward task. This reflects current thinking that someone’s sense of connectedness to a group is relatively fluid and dynamic [11, 16], even in professional environments [21]—and if so, we should be able to proactively address exclusion with the intervention *before* it negatively impacts team outcomes. Again, note that this setup is markedly different from other ostracism interventions that instead seek to reduce *overall* levels of ostracism (e.g., CREW; Leiter et al., 2011), rather than address the negative effects of ostracism as they occur.

Our interventions were inspired by the rich literature on emotion regulation and reappraisal [76–80], which has shown that positively reframing difficult events facilitates more adaptive psychological, physiological, and interpersonal responding [81, 82]. Building on this logic, we reasoned that in organizational contexts, these reappraisals could come in at least three primary forms—it could be an opportunity to learn about the exclusion experiences of other teammates or colleagues; it could be an opportunity to share one’s own exclusion experience with others; or it could be an opportunity to offer more direct feedback on how to change the situation to be more inclusive. Note that in all three cases, the initial negative experience of ostracism is reframed as a positive opportunity for personal growth and development. In Studies 4–5, we designed and tested three exclusion interventions—perspective, mentorship, and empowerment—based on each of the above formulations (see Study 4 for a more detailed treatment).

### Current studies

**Fig 1:**
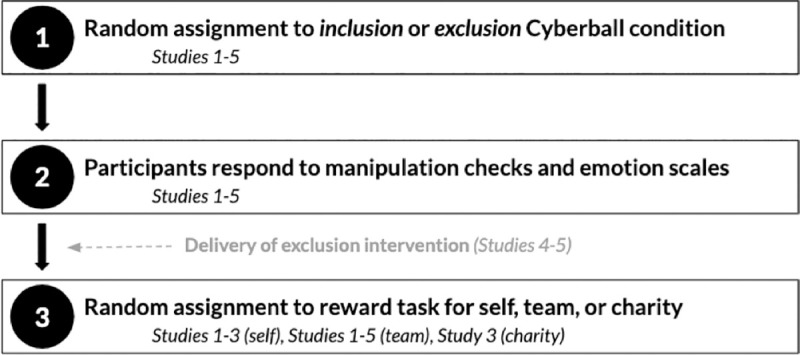
Main experimental steps for studies in the current paper.

This investigation was designed to show that team-based exclusion causes people to work less hard on behalf of their teams, and that select interventions can reduce the negative costs of social exclusion on productivity for one’s team. We used the same general setup for all our studies, which allowed us to cleanly and sequentially examine each of the aforementioned hypotheses (Fig 1).

Overall, we found strong support for Hypotheses 1–3: Excluded participants showed clear antisocial responses, working significantly less hard on behalf of the teammates who excluded them, even if such a response implied sacrificing their own earnings. We also implemented several interventions that successfully mitigated the negative effects of exclusion, and in some cases, even boosted excluded team members’ willingness to work for their team up to or beyond that of included team members.

## Study 1

Study 1 tested the hypothesis that excluded participants would engage in *antisocial* responses, where participants would work less hard on behalf of their team, even at a significant cost to their own earnings.

### Methods

#### Participants

We recruited 298 participants on Amazon Mechanical Turk (*M*_*age*_ = 38.04 years, *SD*_*age*_ = 10.94 years; 125 women). All participants were located in the U.S. (verified by IP address) and compensated for their participation at an average rate of $10 per hour. The study was administered in English and took about 15 minutes to complete. All participants had to meet the following criteria in order to be included in the final sample: (1) 18 years of age or older; (2) currently employed or employed within the past six months; (3) not self-employed; and (4) earning a personal annual income of at least $25,000. In the case of having been employed within the past six months but not currently employed, participants were asked to answer questions as they related to their most recent employment. All participants were recruited using a consecutive sampling design. Note that previous research has shown that Mechanical Turk participants are not only significantly more diverse than typical samples in psychology lab studies [83, 84], but they also provide data of equivalent or greater quality to that of in-lab participants [85]. All studies in the current paper were reviewed and approved by the IRB committee at Ethical & Independent Review Services (www.eandireview.com/) under code #18164 before the studies began. Written informed consent was obtained from all participants across studies.

#### Materials

To examine our main hypotheses, we adapted a version of the online game, Cyberball [58], which has been very widely used in psychological studies on social exclusion and ostracism [86]. During the standard version of Cyberball, participants are told that they will be playing a virtual ball toss game with two other live players. In actuality, the other “players” are bots that are preprogrammed to either *include*, by tossing the ball to the human participant at the same rate that they toss the ball to other players, or to *exclude*, by tossing the ball to the human participant less frequently than to the other players.

With all experiments in this paper, once participants completed a version of the Cyberball manipulation, they moved on to a second task that offered the opportunity for bonus rewards (which was added to their standard compensation upon study completion). Participants were first given a single-digit number, and then on each successive trial, their task was to simply add a new number to the previous total. Participants were told that they had a few seconds to complete each trial, and cash bonuses of $0.01 to $0.25 would periodically be given for correct answers (the total possible reward range represents 2% to 14% of participants’ base compensation for participating). Participants were invited to complete as many trials as they wanted, and before each new trial, participants would indicate whether or not they wished to continue the reward trials or quit and move on. Across experiments, we used this reward paradigm as a measure of participants’ overall task persistence, and combined with our cyberball manipulation, this allowed us to explore how team-based performance was related to social exclusion.

#### Design and procedure

Upon starting the experiment, participants were given an introduction to the Cyberball game, which read as follows:

“Welcome! On the next page, you will be asked to play a game with other volunteers, in real time. After you click ‘Play’, our program will sync up your screen with two other volunteers. This may take a moment, so please be patient. Once everyone is connected, the game will begin, and it will last for a few minutes. If, for some reason, we cannot locate enough other people available at the same time you are online, a message will appear notifying you of this, and you will be directed to the end of the survey where you’ll receive a completion code. You’ll be paid the full amount for your time. Thanks for participating!”

When participants advanced to the next page, they were given a cover story that Cyberball is used to test the effects of mental visualization on performance, along with simple instructions about how to toss the virtual ball to other players. They were instructed to try to mentally visualize the experience (e.g., the appearance and/or personalities of the other players, the environment where the game is being played, etc.), rather than focusing solely on their ball tossing performance. Participants were randomly assigned to either the *inclusion* or *exclusion* condition in Cyberball: In the *inclusion* condition, the bots threw the ball to the participant an equal number of times (i.e., approximately 10 tosses out of 30 total, or ~33%), but in the *exclusion* condition, the bots only threw the ball to the participant once (< 5% of the 30 total tosses).

After completing Cyberball, participants were asked several manipulation check questions, as well as items designed to measure their emotional state. On three of the questions, participants indicated their level of agreement on 5-point scales (1 = *Not at all* to 5 = *Extremely*) with the following statements: *I was ignored; I was excluded;* and *I felt like I belonged*. Participants were also asked to estimate the percentage of total throws they thought they received during Cyberball. Finally, participants were asked to respond to 12 items on the Affect-Adjective Scale [87], which contains six positive emotion items (*happy*, *pleased*, *joyful*, *enjoyment*, *peaceful*, *relaxed*) and six negative emotion items (*anxious*, *angry*, *frustrated*, *depressed*, *unhappy*, *bored*), each of which was rated using 7-point scales (1 = *Not at all* to 7 = *Extremely*).

Next, participants were given an introduction to the reward task with the following instructions:

“Next game! Let’s add some numbers. Here are the rules: You will be given a number to start with. Then you will be given a new number, and you will have a few seconds to add that new number to the previous total. Sometimes you’ll get a cash bonus for adding correctly. Cash bonuses vary from $0.01 to $0.25. You will be notified ahead of time if the current addition task comes with a bonus. After each addition task, a running total of the total compensation you have earned will be displayed (even after additions without a cash bonus).”

Participants were then randomly assigned to a *self-* or *team*-reward condition: In the *self-reward* condition, they were informed they would receive 100% of the earnings in their reward task. In the *team-reward* condition, they were told that they had been randomly selected from the three players in the ball toss game, and any bonus money they earned would be evenly split between them and the other two players from the ball toss game (i.e., they received ⅓ of the bonus money, and the other ⅔ went to their Cyberball teammates). Once participants started the reward trials, they could complete as many trials as they wanted before deciding to end the experiment (the max number of trials was capped at 110).

When participants ended their reward task, they were informed of their total compensation and given a completion code for payment.

### Results

#### Analysis strategy

We employed a 2 x 2 factorial design, with Cyberball condition (2: inclusion vs. exclusion) and Reward condition (2: self vs. team) as between-subject factors. In each experiment (Studies 1–5), our primary DV was the number of trials participants completed during the reward task. Given that the total number of completed trials is actually a proportion of the total number of possible trials, we modeled the DV using an ANOVA with a binomial distribution and logit link function. Significance of main effects and interactions were assessed using Type II likelihood-ratio tests. All post-hoc contrasts were adjusted for multiple comparisons (α = .05) using a Holm correction. For responses to the Affect-Adjective Scale, indices for overall positive emotion and negative emotion were calculated by aggregating participants’ responses for the individual valenced items.

#### Manipulation checks

**Fig 2:**
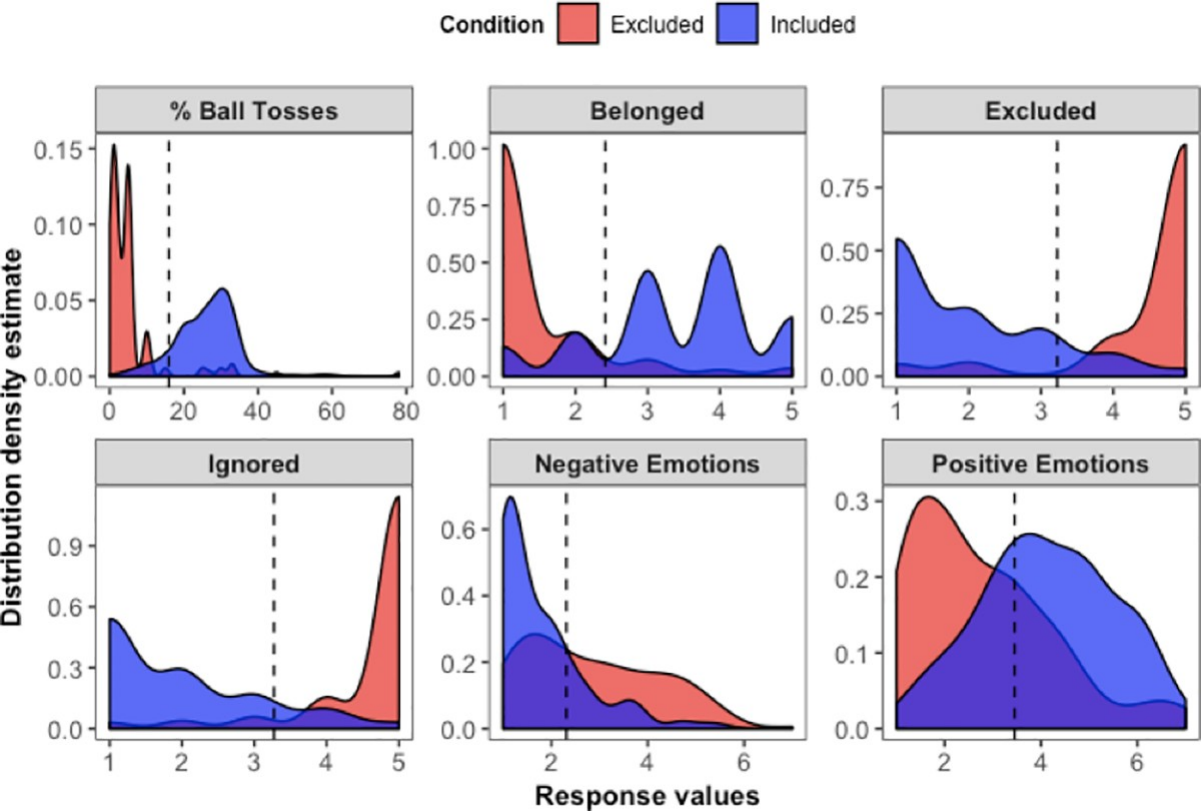
Distributions of manipulation check measures by Cyberball condition in Study 1. Dotted lines show the overall mean response value for each measure. All comparisons by Cyberball condition were significant at *p* < .001. For estimating the percentage of ball tosses, participants typed a number between 0 and 100. For the questions on feelings of belonging, being excluded, and being ignored, participants answered using 5-point scales (1 = *Not at all*; 5 = *Extremely*). For questions on positive and negative emotions, we created composite indices using the individual valenced items from the Affect-Adjective Scale (1 = *Not at all*; 7 = *Very much so*).

Results from our manipulation checks in Study 1 are displayed in Fig 2. Participants in the inclusion condition (compared to participants in the exclusion condition) reported lower scores for feeling ignored (*t*(296) = 23.95, *d* = 2.78, *p* < .001), feeling excluded (*t*(296) = 21.44, *d* = 2.49, *p* < .001), and feeling negative emotions (*t*(290) = 7.72, *d* = 0.91, *p* < .001). Included participants also gave higher scores for feelings of belonging (*t*(296) = 16.65, *d* = 1.94, *p* < .001), positive emotions (*t*(294) = 8.21, *d* = 0.96, *p* < .001), and ball toss percentage estimates (*t*(296) = 19.28, *d* = 2.24, *p* < .001).

#### Reward task

**Fig 3:**
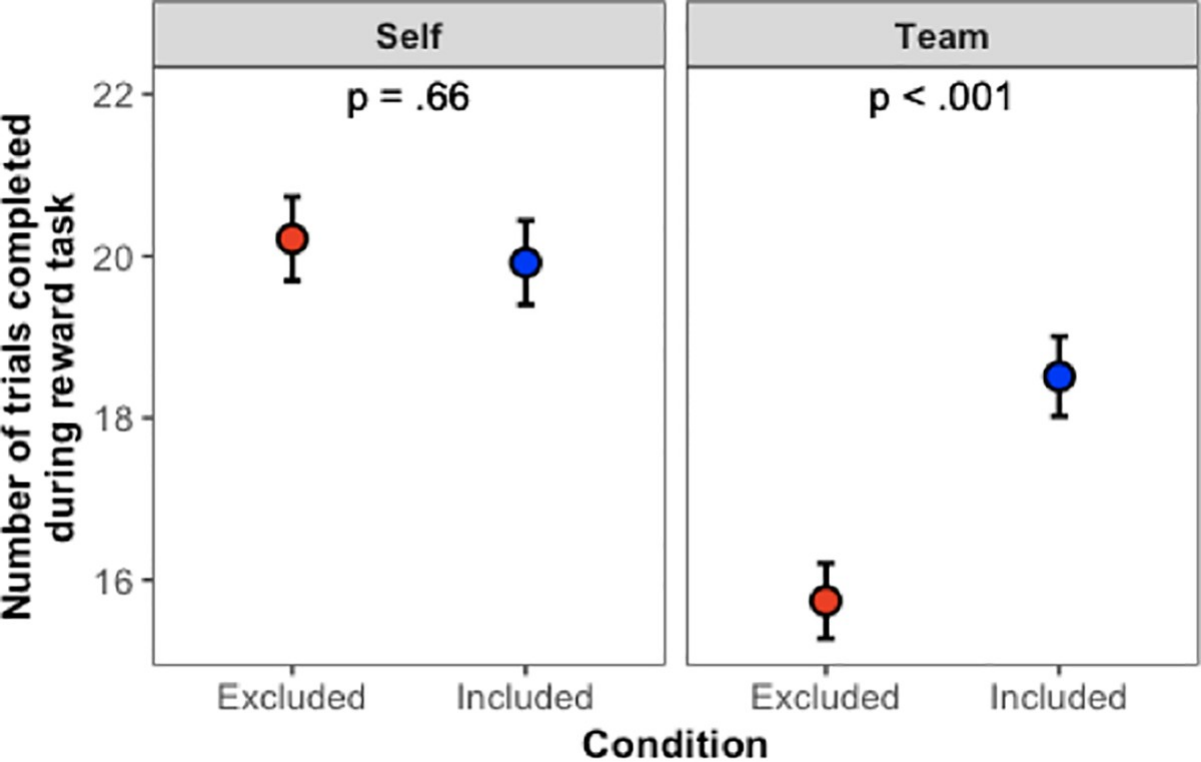
Main results from Study 1. Error bars show ±1 SE.

In a 2 (Cyberball condition: inclusion vs. exclusion) x 2 (Reward condition: self vs. team) binomial ANOVA on the number of trials participants completed during the reward task, all effects were significant. Fig 3 displays the results.

The main effects showed that excluded participants completed fewer trials compared to included participants (χ^2^(1) = 7.41, *p* = .006), and participants completed more trials when earning money for themselves compared to when they were earning money for their team (χ^2^(1) = 41.38, *p* < .001). The Cyberball x Reward interaction (χ^2^(1) = 12.60, *p* < .001) demonstrated that when earning money for their team, excluded participants completed fewer trials than included participants (*z* = 4.44, *p* < .001), but this was not the case when participants were earning money for themselves (*z* = 0.44, *p* = .66). In short, being excluded by one’s teammates significantly affected people’s performance, causing them to work less hard on behalf of their teams, even at some personal cost.

## Study 2

Study 1 revealed strong support for our first hypothesis: Excluded participants worked less hard on behalf of their teams, even if this meant sacrificing their own earnings.

We designed Study 2 to replicate and extend the findings from Study 1, by updating the reward task’s payout structure. In Study 1, rewards on each trial could range from $0.01 to $0.25, regardless of whether participants were assigned to the self- or team-reward task. With this setup, the expected value of the team-reward task is less than that of the self-reward task (since team-reward participants were told they would be sharing the total earnings with their teammates). If participants had attended to this difference in expected value, this could partially explain the main effect of reward condition from Study 1, where regardless of Cyberball condition, participants worked less in the team-reward condition. Note, however, that attention to expected value *cannot* explain the Cyberball x Reward interaction that we observed. Nevertheless, the goal of Study 2 was to replicate our overall effect, while equalizing the expected value in the reward conditions to give us a more precise estimate of people’s willingness to work across conditions.

### Methods

#### Participants

We recruited 245 participants on Amazon Mechanical Turk (*M*_*age*_ = 39.02 years, *SD*_*age*_ = 10.24 years; 121 women). All other details related to participant recruitment and compensation were the same as Study 1.

#### Materials

In Study 2, we used the same tasks and materials as Study 1, with only one change to the expected value of the reward in the reward task, as described below.

#### Design and procedure

Similar to Study 1, when participants were assigned to earn money for themselves, trial rewards still ranged from $0.01 to $0.25. In Study 2, we changed the payouts in the team-reward condition to equalize the expected value. More specifically, to equate the expected value of rewards across conditions, the range of rewards on each trial in the team condition was $0.03 to $0.75 (compared to $0.01 to $0.25 in the self-reward condition). With this setup, even though participants in the team-reward condition would only receive ⅓ of the total rewards, the expected value for the overall amount of reward money they could earn on any trial would be the same as in the self-reward condition (a personal expected value of $0.01 to $0.25 in both conditions).

### Results

#### Analysis strategy

Our analysis strategy in Study 2 was the same as Study 1.

#### Manipulation check

Participants in the inclusion condition (compared to participants in the exclusion condition) reported lower scores for feeling ignored (*t*(243) = 21.53, *d* = 2.76, *p* < .001), feeling excluded (*t*(243) = 22.62, *d* = 2.90, *p* < .001), and feeling negative emotions (*t*(241) = 7.05, *d* = 0.91, *p* < .001). Included participants also gave higher scores for feelings of belonging (*t*(243) = 14.27, *d* = 1.83, *p* < .001), positive emotions (*t*(241) = 8.86, *d* = 1.14, *p* < .001), and ball toss percentage estimates (*t*(243) = 21.42, *d* = 2.75, *p* < .001).

#### Reward task

**Fig 4:**
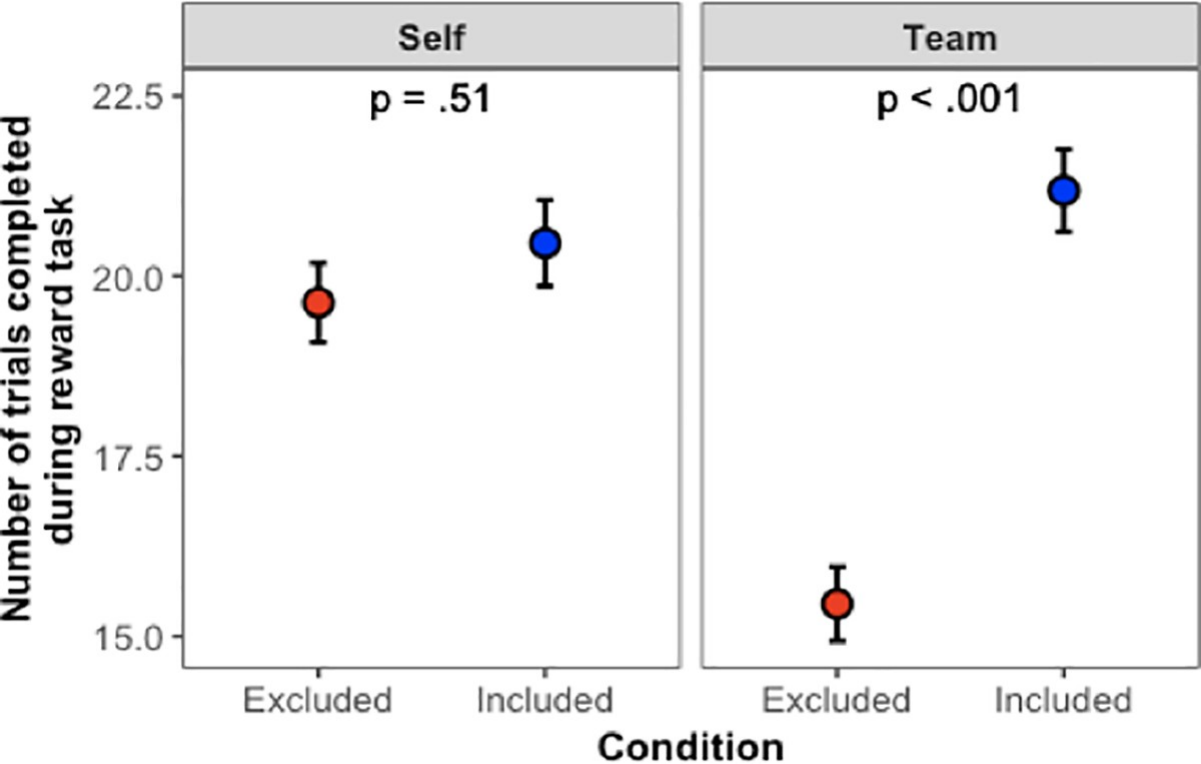
Main results from Study 2. Error bars show ±1 SE.

We replicated the findings from Study 1, as shown in Fig 4. Main effects revealed that excluded participants completed fewer trials than included participants (χ^2^(1) = 41.72, *p* < .001), and participants completed more trials when earning money for themselves compared to when they were earning money for their team (χ^2^(1) = 11.65, *p* < .001). The interaction term (χ^2^(1) = 26.06, *p* < .001) once again demonstrated that when earning money for the team, excluded participants completed fewer trials than included participants (*z* = 8.15, *p* < .001), but this was not the case when participants were earning for themselves (*z* = 1.14, *p* = .51). In short, Study 2 replicated the findings from Study 1.

But what about the effect of equalizing the expected value of the payouts? As revealed by comparing Figs 3 to 4, equalizing the expected value of the payouts appears to have made a difference in how hard people were willing to work for their team. Recall that in Study 1, participants always worked less hard on behalf of their team compared to how hard they worked for themselves, but now that we equalized the expected value of the payouts, participants in Study 2 worked just as hard on behalf of their team as they did for themselves—so long as they were included by their teammates. On the other hand, just as we saw in Study 1, if participants were excluded by their teammates, they worked significantly less hard on behalf of their team, even at a cost to their own earnings.

Indeed, it is remarkable that the team context led participants to work less hard even when it meant earning less money for themselves. Study 1’s finding of reduced effort in the team condition could presumably be a straightforward incentive effect: Working on behalf of the team reduced one’s personal payment. Study 2 eliminated that issue, given that participants could earn identical amounts in the self- and team-reward conditions. As such, Study 2 isolates the *dis*incentive effect of working for one’s team after having been rejected. Rejected persons preferred to earn less for themselves in order to avoid earning money for the team. Such is the powerful antisocial effect of exclusion on team-based performance.

## Study 3

Study 2 replicated the results from Study 1: Excluded participants sabotaged their teams by working less, even if this meant sacrificing their own earnings. Meanwhile, exclusion did not reduce people’s level of work to earn money only for themselves. These effects were also stable across different reward structures, revealing strong evidence for the idea that team exclusion facilitates antisocial responses toward the excluding team members. Collectively, these findings offer substantial support for Hypothesis 1.

Study 3 addressed an important follow-up question: Are the antisocial responses to exclusion specific or general? Recall that in Hypothesis 2, we predicted that the antisocial responses from excluded participants would be specific (i.e., selectively punishing the excluders) rather than general (i.e., punishing members of any other group). To effectively test this in Study 3, we added another reward condition where participants, after being excluded, were asked to earn money on behalf of an unnamed charity (a group that was not responsible for their exclusion). If participants’ antisocial responses are *specific*, excluded individuals should only work less when working for their teammates, with no differences in the self- or charity-reward conditions. If participants’ antisocial responses are *general*, excluded individuals should work less for charity and their teammates alike.

### Methods

#### Participants

We recruited 474 participants on Amazon Mechanical Turk (*M*_*age*_ = 37.97 years, *SD*_*age*_ = 9.89 years; 180 women). All other details related to participant recruitment and compensation were the same as Studies 1–2.

#### Materials

In Study 3, we used the same tasks and materials as Study 2, but with an additional charity condition for the reward task.

#### Design and procedure

Study 3 was the same as Study 2, except for an additional condition where participants were told that they would be sharing money from the reward task with a charitable organization. The reward task prompt for this new charity condition read as follows: “The bonus money you earn will be split between you and a charitable organization—you will receive ⅓ of the bonus money, and the charity will receive ⅔ of the bonus money. The exact charity will be chosen at random from a preselected list of charities after you complete this task. Details on the charity organization will be provided at the end of the survey.” Other instructions and details (including the expected value of rewards across conditions) were the same as Study 2.

**Fig 5:**
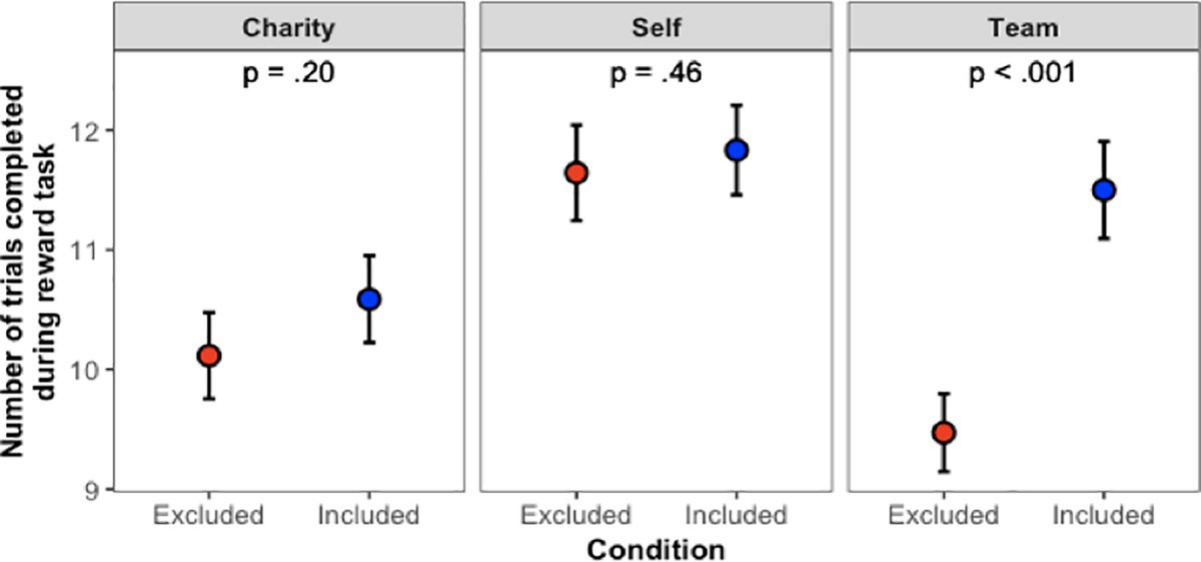
Main results from Study 3. Error bars show ±1 SE.

Note: In Study 3, a technical glitch in the experiment capped participants’ reward trials at a max of 15 (as opposed to Studies 1–2, which were capped at 110), which explains the reduced means in Fig 5. However, note that such a limitation would usually make the effects of the reward task more difficult to detect (due to ceiling effects), but we were still able to replicate the reward task results from Studies 1–2. While we acknowledge this limitation in Study 3, we also believe it (inadvertently) provides further support for the robustness of our findings.

### Results

#### Analysis strategy

Our analysis strategy in Study 3 was the same as Studies 1–2, except that with the added charity condition, our models were run according to a 2 x 3 factorial design, with Cyberball condition (2: inclusion vs. exclusion) and Reward condition (3: self vs. team vs. charity) as between-subject factors.

#### Manipulation check

As in Studies 1–2, participants in the inclusion condition (compared to participants in the exclusion condition) reported lower scores for feeling ignored (*t*(472) = 33.91, *d* = 3.12, *p* < .001), feeling excluded (*t*(472) = 33.28, *d* = 3.06, *p* < .001), and feeling negative emotions (*t*(464) = 10.35, *d* = 0.96, *p* < .001). Included participants also gave higher scores for feelings of belonging (*t*(472) = 22.80, *d* = 2.10, *p* < .001), positive emotions (*t*(466) = 10.49, *d* = 0.97, *p* < .001), and ball toss percentage estimates (*t*(472) = 33.14, *d* = 3.05, *p* < .001).

#### Reward task

There are two important takeaways from the Study 3 results, which are shown in Fig 5. First, we replicated both main effects from Studies 1–2. These main effects revealed that excluded participants completed fewer trials than included ones (χ^2^(1) = 30.45, *p* < .001), and participants tended to complete more trials when earning money for themselves than for their team (χ^2^(2) = 61.32, *p* < .001). Second, we also replicated and extended the key interaction observed in Studies 1–2. As before, the interaction (χ^2^(2) = 22.46, *p* < .001) revealed that when earning money for their team, excluded participants completed fewer trials than included participants (*z* = 7.04, *p* < .001), but this decrease in performance was not the case for participants who were earning money for themselves (*z* = 0.74, *p* = .46) or for participants who were earning money on behalf of a charity (*z* = 1.66, *p* = .20).

In other words, as predicted in Hypothesis 2, people’s reactions to being excluded were *specific*: Excluded individuals worked less hard on behalf of the teammates who had excluded them, but being excluded did not affect how hard people were willing to work for themselves or how hard people were willing to work on behalf of an unrelated group (charity).

## Survival analysis for Studies 1–3

Note that our main outcome of interest in Studies 1–3 is *when* something happens—the time (trial) at which participants decide to stop the reward task. This empirical scenario is uniquely suited to survival analysis, a method focused on evaluating the time until an event occurs (i.e., time-to-event analysis; [88, 89]). Survival analysis is popular across a wide array of fields, including epidemiology (e.g., recurrence of disease; [90]), economics (e.g., unemployment duration; [91]), engineering (e.g., machine failures and industrial design; [92]), and psychology (e.g., onset of mental health issues; [93]). Here, we combined all data from Studies 1–3 to run a survival analysis on the time at which participants chose to discontinue the reward task. The main benefit of this analysis is that it allows us to precisely estimate the consequences of exclusion on team-based performance. Specifically, this analysis will allow us to answer the following question about our paradigm: When people are excluded by their teammates, how much does this increase their risk of dropping out and no longer persisting at the reward task?

Survival analysis data typically consists of three main components: (1) the time until the event of interest occurs for each participant (i.e., the number of reward trials each participant completed before stopping); (2) any grouping information for the participants (i.e., participants’ Cyberball [included vs. excluded] and reward task [self vs. team] conditions); and (3) the censoring information for each participant. *Censoring* refers to situations where the event of interest did not occur during the time we observed the participant, but we cannot be certain the event would never have occurred. For example, in a longitudinal study on mental health, you might be tracking children predisposed to a certain disorder to gauge whether they develop symptoms (and if so, the age of symptom onset). However, for some participants, you might lose contact with them at some point, without knowing about any potential symptom onset; therefore, we cannot know if those participants ever developed symptoms, and if so, when. In this example, survival analysis allows censored observations to contribute to the outcome of interest up to the time they ceased to be followed—so the main advantage is that data can be unbalanced across participants. With our studies, censoring only applies to Study 3, where a technical issue capped participants’ max number of reward trials at 15; thus, for Study 3 participants that completed 15 trials, we do not know when they would have stopped the reward task if the trials were conducted using the standard 110-trial cap from the other studies. To address this, all Study 3 participants that completed the max number of 15 reward trials were censored, in order to not influence estimates for other participants.

Using this data, we fit a survival function across reward trials—in our case, the probability that participants will continue in the reward task. Across reward trials, we obtain the probability that a participant would exceed that trial number; therefore, over time (and reward trials), the survival function will go down, indicating reduced probability that participants would continue the reward task as trial numbers increase.

Overall, when participants were earning rewards on behalf of their team, included participants (compared to excluded participants) had higher survival probabilities across reward trials (χ^2^(1) = 11.03, *p* < .001). In contrast, when participants were earning rewards for themselves, there was no difference between the survival functions for included vs. excluded participants (χ^2^(1) = 0.16, *p* = .69). To confirm the interaction between Cyberball condition (included vs. excluded) and Reward condition (self vs. team), we also ran Cox proportional hazard models with both factors included. We confirmed the significance of the interaction using a likelihood-ratio test that compared models both with vs. without the interaction term (χ2(1) = 3.91, p = .048). Excluded participants were at a greater risk of dropping out and stopping work on their task.

But exactly how much of a greater risk? We derived an index for the relative risk between conditions by running Cox proportional hazard models, also referred to as a hazard ratio (HR; [94, 95]). Within the team-reward condition, our analysis revealed that excluded participants were at 1.42 times the risk of dropout compared to included participants (i.e., *HR = exp(β) = exp(0.3491) = 1.42*, 95% CI [1.15, 1.75], *p* < .001). We did not observe any such differences in the self-reward condition, where excluded participants were only at 1.05 times the risk of dropout compared to included participants (HR = 1.05, 95% CI [0.84, 1.29], *p* = .69). Overall, this re-analysis of the data from Studies 1–3 clearly and succinctly demonstrates the effects of exclusion on team-based performance.

## Study 4

We found strong support in Studies 1–3 for Hypotheses 1 and 2: Excluded participants worked less for their teams than for themselves, even if this meant sacrificing their own earnings (Hypothesis 1). This sabotage effect was also specific, meaning excluded individuals selectively punished teammates from the excluding group but not an unrelated group (Hypothesis 2).

In Study 4, we moved on to test hypotheses about which interventions (if any) might be effective in mitigating the negative effects of exclusion on participants’ willingness to work for their teams. We designed and tested three new interventions, which were largely inspired by the literature on emotion regulation [78, 96, 97]. Emotion regulation strategies are crucial in promoting more adaptive psychological, physiological, and interpersonal responding to social stressors [76]. There are many ways one can regulate their emotions while coping with negative events, including distraction and emotional suppression [78, 82, 98, 99], but reappraisal is among the most effective strategies [78, 80, 100]. Reappraisal occurs when an initially negative emotional event (e.g., social exclusion) is cognitively reframed in terms of potential positive benefits (e.g., personal growth; [76, 77, 96, 97]). While there is limited research on how such strategies could extend to coping with ostracism, studies that have implemented similar approaches have shown promising results [81, 82].

While our intervention designs were originally inspired by the emotion regulation literature, it is worth noting some key differences between our interventions and prior reappraisal strategies. First, emotion regulation strategies usually involve the activation of a goal to influence the trajectory of one’s emotions [101]. For example, one could have the goal of regulating their sadness in order to simply feel less sad or in order to appear more composed during a social interaction. Such goals are often conscious (as with trying to inhibit laughter to inappropriate material), but they can also be implicitly engaged outside of conscious awareness (where one might quickly turn away from upsetting content; [102]). Second, it is an active area of research about whether emotion regulation strategies are most effective before, during, or after the emotion-inducing event (often referred to as *regulatory timing*; [103, 104]). Research has shown that reappraisal may be most effective in situations where it is proactively implemented before the distressing event [105, 106], though this is not always the case [107]. We offer a more in-depth evaluation of our interventions in light of these topics in the *General Discussion*, but our main goal with these interventions was not to enact a traditional reappraisal induction, as with studies that explicitly tell participants to reappraise [105]. Rather, we aimed to *adapt* the reappraisal approach (i.e., cognitively reframing distressing events in terms of potential benefits) into different strategies that would help people to cope with the negative consequences of exclusion in more applied settings (regardless of regulatory goals or timing).

With our studies, we reasoned that in organizational contexts, these reappraisals could come in at least three primary forms, which we operationalize below:

1. ***Perspective*:** Does learning about others who have experienced social exclusion prompt people to reappraise their own exclusionary experiences? Indeed, prior work in applied psychology has shown that reading perspectives or testimonials from similar others can increase feelings of social belonging [70, 71]. Perspective-taking might also function as a sort of self-distancing after a negative event, allowing one to view the situation through a more objective lens [108, 109]. This also aligns with other findings showing that people have a tendency to seek out others’ perspectives after rejection [110], and even small reminders of social activity and connection can reduce aggressive responses following ostracism [48]. We adapted this idea to our experimental paradigm, where excluded individuals would read about previous participants’ experiences playing Cyberball in our studies.
2. ***Mentorship*:** Asking people to give advice might be another way to get people to reappraise their experience. For example, giving advice about how to deal with exclusion could actually help the advice-giver (the excluded participant) deal with their own exclusion. Giving advice is a crucial part of being an effective mentor [111–113]. Moreover, the process of being a mentor and giving advice to others leads to increases in one’s own sense of power and control [114], growth in personal and professional development [115–117], and feelings of interpersonal closeness [118, 119]. We incorporated these ideas into our experiments by asking excluded participants to act as a mentor by offering advice and insights that would make future participants feel better after being excluded.
3. ***Empowerment*:** A final way to get people to reappraise their experience might be to offer them an opportunity to give more explicit feedback about how to improve the situation. Here, the negative effects of exclusion could be resolved by instilling a sense of empowerment—or the perceived ability to enact meaningful change. At work, empowerment leads to a greater job satisfaction [120, 121], positive health outcomes [122, 123], and a boost in resilience [124]. Empowerment may also help the excluded individual regain a sense of control [125, 126], which has been shown to be a fundamental psychological need associated with social belonging [9]. We integrated these ideas into our studies by having excluded individuals write about what they would do if they had the power to change the Cyberball game to make it more inclusive and enjoyable.

Recall that in Hypothesis 3, we predicted that once a participant has been excluded, their persistence in the team-based reward task would be optimized (i.e., returned to the level of other *included* participants) if they engaged in an applied intervention. At this point, we did not have any predictions for which interventions would be most effective, but we return to this question in Study 5.

### Methods

#### Participants

We recruited 295 participants on Amazon Mechanical Turk. All other details related to participant recruitment were the same as Studies 1–3.

#### Materials

We used the same materials as Study 2, with three substantial changes made to the design and procedure.

#### Design and procedure

Given our focus on exclusion interventions, we planned on only analyzing excluded participants (we also collected 290 additional participants in Study 4 that were randomly assigned to the inclusion condition in Cyberball and also received the interventions. Given that we were mainly concerned with the effects of the intervention on excluded individuals, results for Study 4 inclusion participants are reported in the S1 File). As our interventions were specifically designed to mitigate the negative effects of exclusion in a team-based setting, we also dropped the self-reward condition (i.e., all Study 4 participants were told they were earning rewards on behalf of their team in the reward task). We randomly assigned participants to one of three interventions (or a fourth control condition) immediately after finishing Cyberball:

1. ***Perspective***: Participants in the *perspective* condition read about the experience of a previous participant who played Cyberball. These perspectives were sourced from actual prior participants who were asked to write about their experience being excluded while playing Cyberball in our experiment. (e.g., “I received the ball a few times … The outcome of the game made me try harder to prove that I am worthy later on in the survey. This game reminded me of teamwork situations in real life. When I am dealing or interacting with others, I try to be as fair to everyone as possible.”)
2. ***Mentorship***: Participants in the *mentorship* condition were asked to write something for future Cyberball players to make them feel better if they were excluded. The instructions read as follows: “When people play the ball toss game you just did, someone often feels excluded. Imagine you were to talk to someone who has just been excluded from the game. Please write a short paragraph describing what would you say or do for them to make them feel better.”
3. ***Empowerment***: Participants in the *empowerment* condition were asked to come up with solutions for how to improve the Cyberball experience. The instructions read as follows: “Based on your experience playing this ball toss game, please write about how you would change the game if you could? What would you fix to make the game more enjoyable? For example, how would you ensure that everyone feels included?”
4. ***Control***: In Study 4, we also added a control condition to ensure that any observed effects were not simply a byproduct of engaging in another task before the reward trials; our control condition was designed to closely mirror the setup of the perspective condition but without the targeted focus on Cyberball. Participants in the control condition also read perspectives from previous Cyberball participants, but these perspectives did not focus specifically on people’s experience playing Cyberball; rather, these control perspectives were about people’s experience with other unrelated topics (i.e., what they learned from participating in science experiments on MTurk, etc.) (e.g., “Playing this game actually made me think a lot about how experiments are designed. I realized I’ve actually learned a lot about how experiments work from just doing them on Mturk. I guess this is how science comes to life. Pretty interesting actually.”). We expected participants in the perspective, mentorship, and empowerment interventions to show improved performance compared to participants that were assigned to this control condition.

### Results

#### Analysis strategy

Our analysis strategy in Study 4 was similar to Studies 1–3, except that with the changes in study design, our models were run with Intervention (4: perspective vs. mentorship vs. empowerment vs. control) as the only between-subject factor.

#### Manipulation check

**Fig 6:**
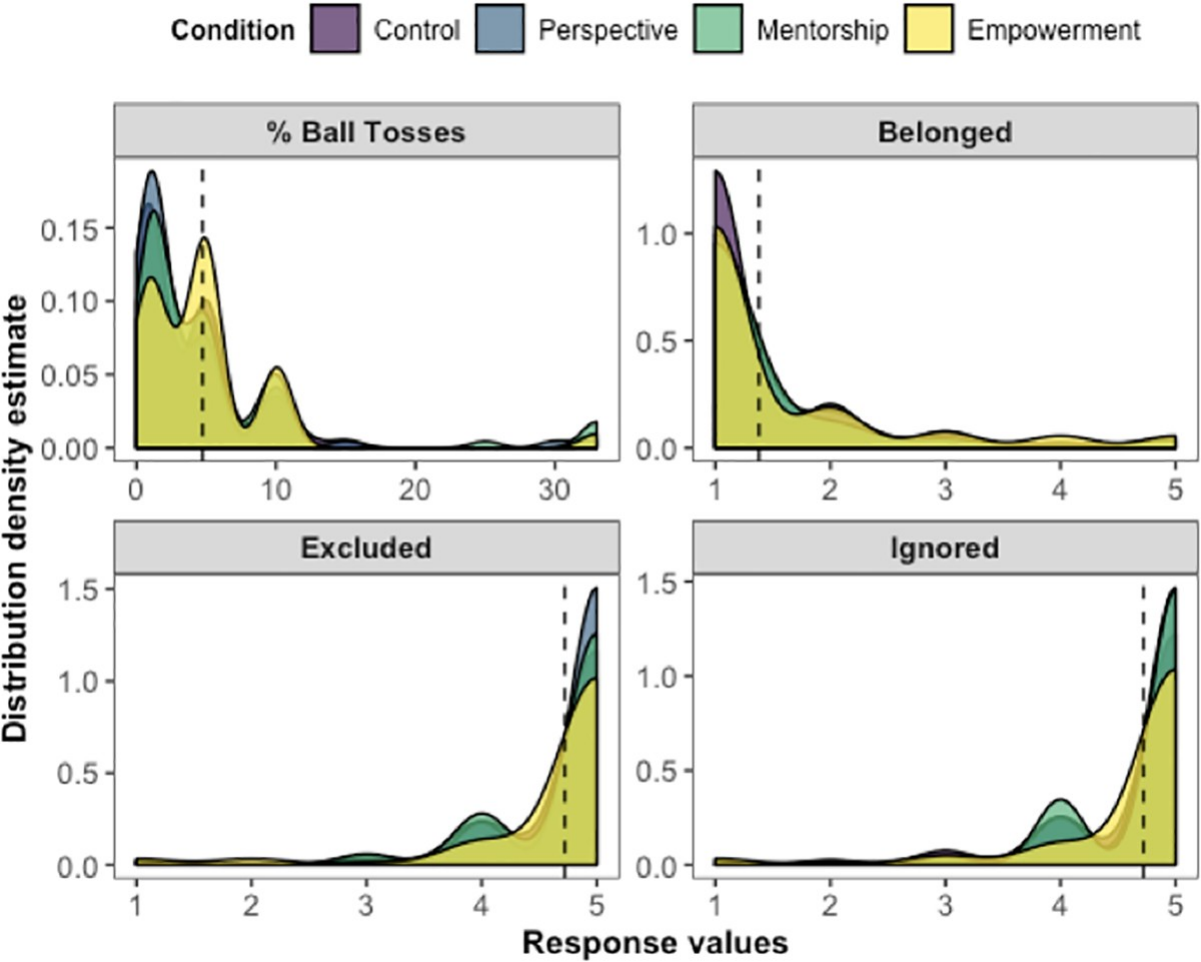
Distributions of manipulation check measures by intervention condition in Study 4. The main participants in Study 4 were only assigned to the exclusion condition in Cyberball. Dotted lines show the overall mean response value for each measure. There were no differences by intervention group for any of the measures. For estimating the percentage of ball tosses, participants typed a number between 0 and 100. For the questions on feelings of belonging, being excluded, and being ignored, participants answered using 5-point scales (1 = *Not at all*; 5 = *Extremely*).

To ensure that participants in all intervention conditions experienced similar degrees of exclusion during Cyberball, we analyzed the manipulation check measures with the prediction that there would be no significant differences between intervention groups. Our prediction was confirmed. We did not observe any differences by intervention group on feeling ignored (*F*(3, 291) = 0.32, *p* = .81, η^2^ = .003), feeling excluded (*F*(3, 291) = 0.37, *p* = .78, η^2^ = .004), feelings of belonging (*F*(3, 291) = 0.98, *p* = .40, η^2^ = .01), or estimates of ball toss percentage (*F*(3, 291) = 0.74, *p* = .53, η^2^ = .008). Fig 6 displays these results.

#### Reward task

**Fig 7:**
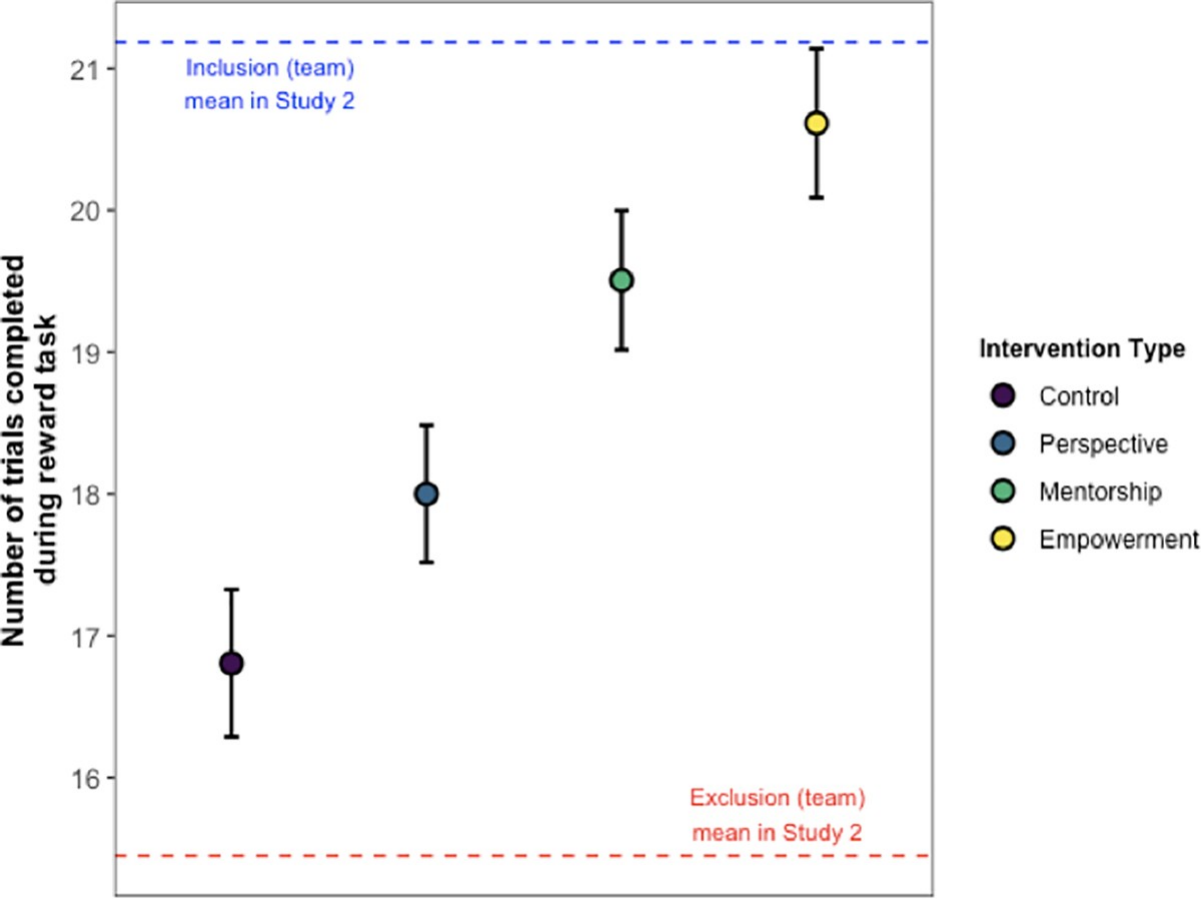
Main results from Study 4. The number of completed reward tasks is shown on the *y*-axis, and intervention conditions are color-coded. The blue dotted line shows the grand mean reward task completion for all *inclusion* participants in Study 2 that were earning rewards on behalf of their *teams*. The red dotted line shows the grand mean reward task completion for all *exclusion* participants in Study 2 that were earning rewards on behalf of their *teams*. Grand means for Study 2 inclusion and exclusion participants are shown as a reference, given the similarity in task design and reward structure. Error bars show ±1 SE.

Fig 7 displays the results from Study 4, which revealed a main effect of Intervention (χ^2^(3) = 37.5, *p* < .001). Further post-hoc analyses revealed that the interventions differed in the magnitude of their effectiveness, compared to controls. Indeed, participants in the perspective condition completed more reward trials than controls, but this difference was not significant (*z* = 1.82, *p* = .14). By contrast, participants who received the mentorship intervention displayed significantly greater levels of task persistence compared to controls (*z* = 4.09, *p* < .001) and perspective participants (*z* = 2.40, *p* = .05). Similarly, participants who received the empowerment intervention also completed more reward trials than controls (*z* = 5.60, *p* < .001) and perspective participants (*z* = 4.04, *p* < .001). The mentorship and empowerment conditions were not significantly different from one another (*z* = 1.71, *p* = .14). These results provide initial evidence that it is possible to reduce the negative effects of social exclusion on team-based performance. Our mentorship and empowerment interventions delivered the most promising results.

## Study 5

Study 4 demonstrated that multiple interventions were able to lessen the negative effects of exclusion on team-based performance. This offers preliminary support for Hypothesis 3.

In Study 5, we aimed to replicate and extend our intervention findings. More specifically, in Study 4, we did not have clear baseline comparison conditions for included or excluded participants who were not treated with an intervention. We added these conditions in Study 5, in addition to the three intervention conditions, which allowed us to compare the effectiveness of the interventions to baselines of inclusion and exclusion. Also, in Study 4, we observed an ordering in the effectiveness of the interventions—where perspective was less effective than mentorship, which was about as effective as empowerment. We did not predict this ordering beforehand, so we not only designed Study 5 to allow for replication, but we also preregistered all methods and analyses in Study 5 on the Open Science Framework (https://osf.io/) to confirm our initial findings from Study 4.

### Methods

#### Preregistration

We preregistered all of our hypotheses, methods, and analyses for Study 5 prior to data collection. This finalized preregistration report can be accessed here: https://osf.io/djwa7/?view_only=0844cd7c6dbb440c86292cd332a76550.

#### Participants

We recruited 502 participants on Amazon Mechanical Turk (*M*_*age*_ = 35.39 years, *SD*_*age*_ = 10.32 years; 223 women). All other details related to participant recruitment and compensation were the same as Studies 1–4. Our target sample size of *n* = 500 (100 participants per intervention condition) was based on a power analysis where we aimed for more than .95 power to detect a small-to-medium effect size of *f* = .2 (alpha = .05).

#### Materials

We used the same materials as Study 4, but we added baseline inclusion and exclusion conditions, which we detail below.

#### Design and procedure

In Study 5, we adapted Study 4 to include inclusion and exclusion baseline conditions (where participants did not receive any intervention; instead, these participants were simply included or excluded by their teammates and then asked to work on behalf of their team). Note that these baseline conditions are essentially equivalent to the primary conditions of interest in Studies 1–3 but having them here in Study 5 will allow us to better understand the effectiveness of our interventions.

### Results

#### Analysis strategy

Our analysis strategy was similar to Study 4, except that with the added conditions, our models in Study 5 were run with more levels in the Condition between-subject factor (5: exclusion control, inclusion control, perspective, mentorship, empowerment).

#### Manipulation check

**Fig 8:**
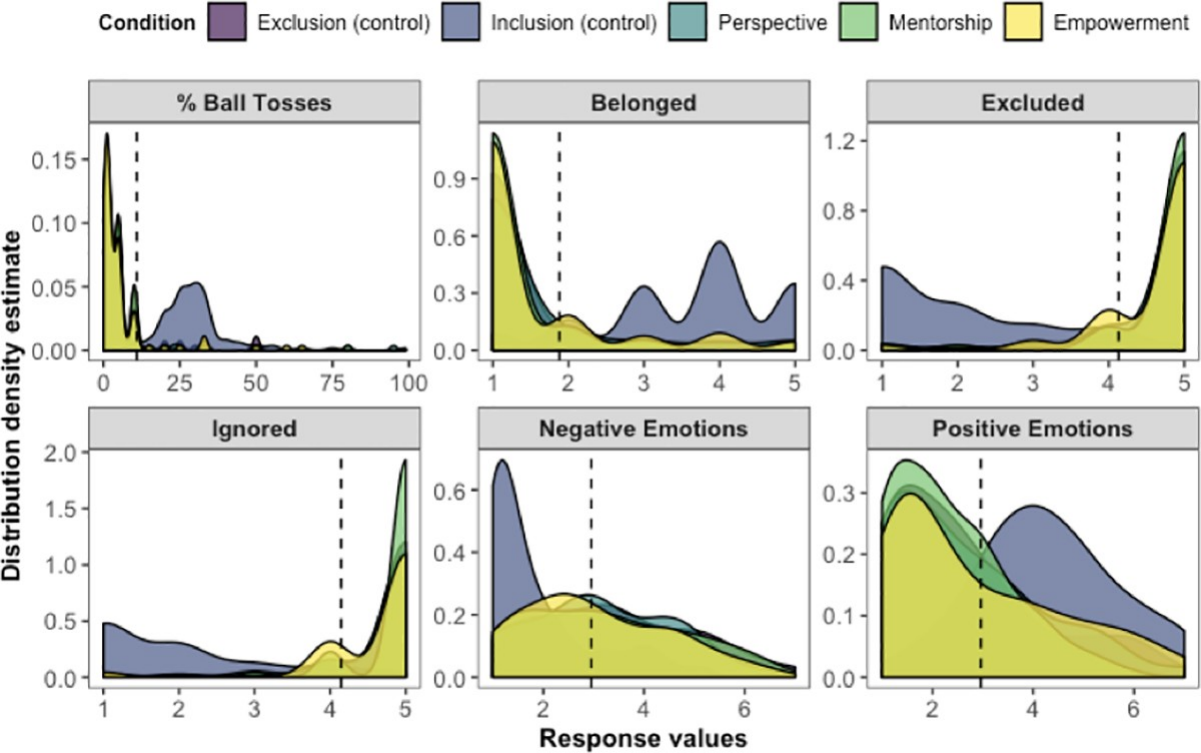
Distributions of manipulation check measures by condition in Study 5. Dotted lines show the overall mean response value for each measure. All main effects by condition were significant at *p* < .001. For estimating the percentage of ball tosses, participants typed a number between 0 and 100. For the questions on feelings of belonging, being excluded, and being ignored, participants answered using 5-point scales (1 = *Not at all*; 5 = *Extremely*). For questions on positive and negative emotions, we created composite indices using the individual valenced items from the Affect-Adjective Scale (1 = *Not at all*; 7 = *Very much so*).

Manipulation check results from Study 5 are displayed in Fig 8. Main effects of condition across all measures were significant (*F*s(4, 497) ≥ 18.76, *p*s < .001, η^2^s ≥ .13). We confirmed that when comparing inclusion control vs. all other excluded participants (exclusion control, perspective, mentorship, and empowerment conditions), inclusion led to greater feelings of belonging (*F*(1, 500) = 374.69, *p* < .001, η^2^ = .43), positive emotions (*F*(1, 500) = 109.06, *p* < .001, η^2^ = .18), and ball toss percentage estimates (*F*(1, 500) = 350.96, *p* < .001, η^2^ = .41), along with reduced levels of feeling ignored (*F*(1, 500) = 865.46, *p* < .001, η^2^ = .63), feeling excluded (*F*(1, 500) = 757.15, *p* < .001, η^2^ = .60), and negative emotions (*F*(1, 500) = 71.85, *p* < .001, η^2^ = .13). There were no differences on any of the manipulation check measures among participants in the exclusion conditions (*F*s(3, 395) ≤ 2.02, *p*s ≥ .11, η^2^s ≤ .015).

#### Reward task

**Fig 9:**
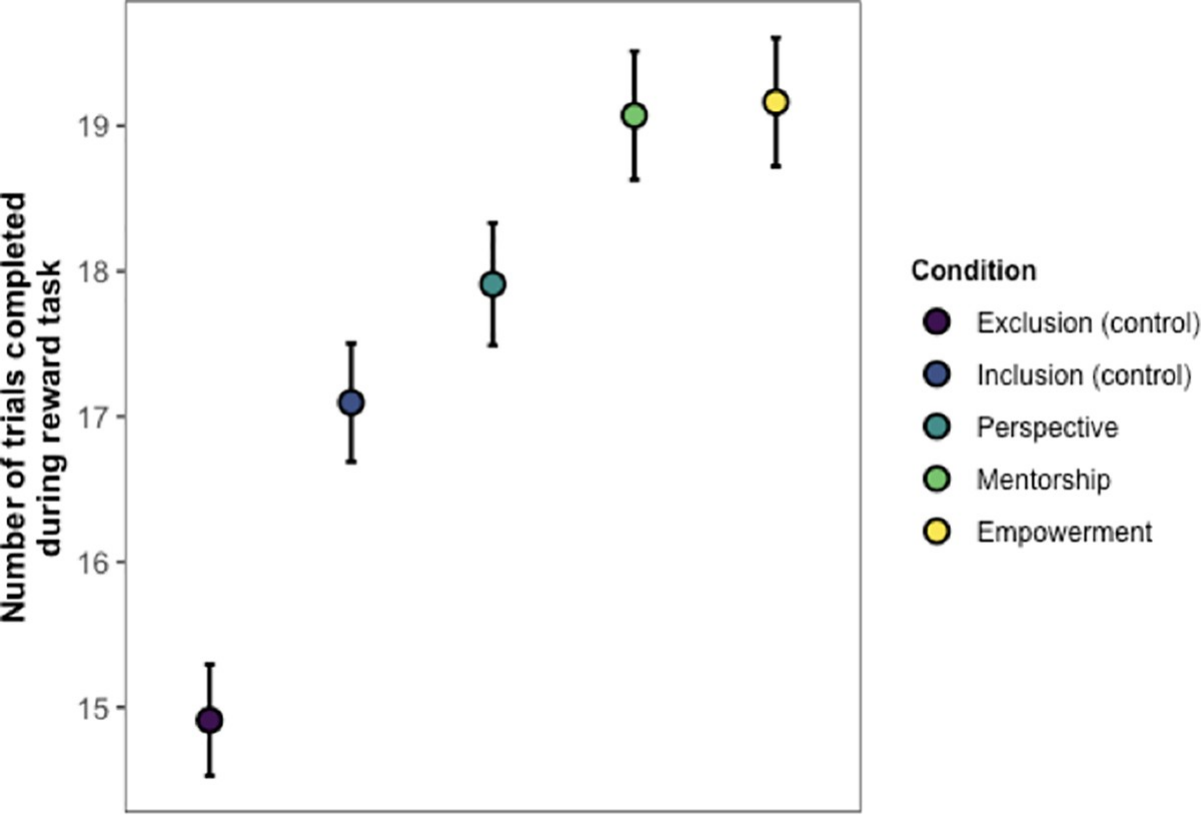
Main results from Study 5. Error bars show ±1 SE.

Fig 9 shows the main findings from Study 5, which revealed a main effect of Condition (χ^2^(4) = 84.4, *p* < .001). We replicated the main pattern of results from Studies 1–3, where participants who were excluded by their team completed significantly fewer reward trials compared to participants who were included by their team (*z =* 4.23, *p* < .001). Once again, social exclusion led to a significant decrease in team-based performance.

But were our interventions successful in reducing these negative costs? To test the effectiveness of our interventions, we first compared the performance of participants who were excluded (but then received an intervention) to exclusion control participants, who were simply excluded and received no intervention. All three of our interventions—perspective, mentorship, and empowerment—proved effective, significantly increasing participants’ task persistence above that of excluded participants who did not receive any intervention (*z*s *≥* 5.71, *p*s < .001).

We also addressed the question of whether our interventions could boost excluded individuals’ performance to the level of included individuals (or even beyond). We did this by comparing the intervention conditions to each other, as well as to the inclusion control condition. All three of our interventions increased participants’ task persistence to or beyond the level of inclusion controls. More specifically, participants in the perspective condition did not differ from inclusion controls in the number of completed reward trials (*z* = 1.52, *p* = .26). However, participants who received the mentorship and empowerment interventions completed significantly *more* reward trials than inclusion controls (*z*s *≥* 3.60, *p*s < .002). The mentorship and empowerment conditions were not significantly different from one another (*z* = 0.16, *p* = .87). Note that these results revealed a similar ordering as Study 4 for the effectiveness of the interventions, where mentorship and empowerment had the greatest impact.

In sum, people who were excluded by their team worked less hard on behalf of their team. To remedy these negative effects of social exclusion, we designed three interventions based around the ideas of perspective-taking, mentorship, and empowerment. All three interventions were effective in boosting persistence in the team-based reward task, with some of our interventions (i.e., mentorship and empowerment) not only erasing the negative effects of exclusion on task persistence, but even boosting excluded participants’ performance beyond that of other *included* participants.

## General discussion

We sought to demonstrate both the antisocial effects of being socially excluded and the remedial effects of some possible interventions. Our findings across five studies are clear and robust: Excluded participants worked less hard for their teams (who had excluded them), even if this meant sacrificing their own earnings (Hypothesis 1). Indeed, in Study 2, people were paid equally for their work regardless of whether they were working for themselves or for their team. But when excluded by their teammates, people reduced their efforts substantially. Apparently, people would rather earn less money themselves than benefit those that excluded them. Moreover, these antisocial responses were socially specific: Excluded workers selectively punished teammates from the excluding group but not an unrelated group (Hypothesis 2). In Study 3, for example, people’s willingness to work for an unnamed charity organization did not change as a function of whether they had been rejected by their team. We also devised three intervention strategies to reduce the negative cost of social exclusion on people’s willingness to work for their teams. In all cases, these interventions were successful at improving persistence in the team-reward task, and in some cases (i.e., mentorship and empowerment), we even managed to boost excluded participants’ performance beyond that of other *included* participants (Hypothesis 3). These studies add novel insights to a variety of fields that have examined the consequences of social exclusion, including social psychology, organizational behavior, and management science.

Perhaps most notably, our intervention results (Studies 4–5) speak directly to recent calls for evidence-based interventions to combat exclusion [68, 69]. Moreover, our findings are especially relevant for better understanding antisocial workplace behaviors [21, 49, 50], and how their associated performance costs might be mitigated. These interventions are also novel in their design and application, seeking to reactively mitigate the negative effects of ostracism immediately after it has occurred (compared to other more proactive interventions that try to increase *overall* levels of civility over the course of many months; [127]). Obviously, we agree that proactively addressing ostracism at work is critical; however, we also believe that some level of ostracism is inevitable [10, 11, 16, 73], so interventions are needed to help people cope with those situations as they arise. Not only do our findings show that such reactive ostracism interventions improve team outcomes, but we also offer three specific interventions that can be readily applied in work settings.

Consider that we observed a clear difference in intervention effectiveness, where the mentorship and empowerment interventions were particularly successful. Based on the available literature, we believe that reappraising ostracism as mentorship and empowerment opportunities may have more powerful restorative effects on the psychological needs of excluded individuals (i.e., control and belonging; [30]). In other words, asking excluded individuals to give advice (mentorship) or be involved in strategic change (empowerment) might allow them to regain a sense of control and belonging in potentially threatening situations [37, 128], thereby reducing the negative costs of social exclusion in team settings. Future research might examine this mechanistic account of our interventions, and hopefully put these insights to use in designing inclusion-based initiatives for teams in the workplace.

As mentioned previously, our interventions were inspired by the emotion regulation literature on reappraisal [76–80, 96, 97], but this does not mean that our interventions were intended to replicate traditional reappraisal manipulations. More specifically, we did not give our participants an explicit reappraisal goal during their intervention, whereas previous studies often give participants direct instructions on how to rethink and reframe the emotional event [105, 106]. Another difference is that our interventions occurred after the emotion-inducing event (i.e., exclusion), whereas many other traditional reappraisal strategies are thought to be antecedent-focused (i.e., occurring *before* the stressor; Gross, 2001). However, other studies have demonstrated reappraisal-based strategies to be effective both before *and* after emotion-inducing events [107], so there is some flexibility in the application of these interventions. Overall, while we recognize the importance of these distinctions, our main goal with these interventions was to *adapt* (rather than replicate) the reappraisal approach, in giving participants multiple concrete strategies to cognitively reframe ostracism in terms of its potential positive benefits. In doing so, the results from Studies 4–5 clearly show that these reappraisal-based interventions were effective in helping people to cope with the negative consequences of exclusion.

It is particularly informative to consider our results in light of the chronology of how social exclusion typically occurs, which can be loosely categorized into three stages: *event* (the manner in which someone gets excluded), *feelings* (how someone feels upon being excluded), and *consequences* (the downstream behaviors that follow exclusion). Regarding the first *event* stage, our exclusion manipulation was implemented through Cyberball [58], and our participants were assigned to teams. Cyberball is a very popular exclusion paradigm as it is a minimal manipulation of ostracism (which may also affect feelings of meaningfulness, anxiety, and control; [11]), and the task also lends itself to online studies. There are many other manipulations of social exclusion [11], including the “life alone” manipulation (where rejected participants are given false feedback that they are the type who will end up alone later in life; [43, 129]), the “get acquainted” manipulation (where rejected participants are told no one wants to work with them after a group discussion; [130]), public goods dilemma games [131], conversational role plays (e.g., chat rooms, text messages, and face-to-face interactions; [12, 132–134]), and imagination prompts to “relive” rejection experiences [31, 44]. The frequent use of all of these exclusion manipulations likely contributes to discrepant findings because they differ in various minor ways (which in turn affects the manner and timecourse for how the exclusion is interpreted; [9]). Thus, while our results are strong and reliable, they should still be extended to other exclusion manipulations and team setups.

This leads to the second stage, *feelings*: How does someone feel upon being excluded? There are two prominent frameworks that have been proposed—an *emotion-based* account and a *numbness* account. The emotion-based account posits that responses after exclusion are primarily driven by changes in mood and negative emotion, which can alter or frustrate other basic psychological needs (e.g., belonging, control, etc.; [9]), but the exact structure for this chain of psychological events is still up for debate [8]. This idea is consistent with studies demonstrating that exclusion can mimic neural responses to physical pain (e.g., increased activation in the dorsal anterior cingulate cortex; [135, 136]) and cortisol responses after threat [137]. Many other studies have also found higher levels of self-reported distress following ostracism, with large effect sizes and positive linear relationships with the amount of experienced ostracism [11, 137–139]. In contrast, the numbness account posits that social exclusion causes a temporary absence of affect, rendering the person relatively numb to both emotional and physical pain [43, 129, 140]. This idea helps explain other “freezing” responses that have been reported after exclusion, like the perception of time standing still, a heightened sense of meaninglessness, higher pain thresholds, flat affect, and increased lethargy [141, 142].

Overall, our results appear consistent with the emotion-based account: We consistently found that exclusion both increased negative emotions and reduced positive emotions (see Figs 2, 6 and 8). Many of our other findings are also consistent with this account. For example, research has shown that antisocial responses to rejection may serve as attempts to rebalance thwarted psychological needs, such as the need for control [9, 37]. Likewise, replenishing these thwarted psychological needs can reduce aggressive after-effects of social exclusion [47, 48]. This could also explain why our mentorship and empowerment interventions (i.e., “active” interventions that likely allow for a sense of regaining control) outperformed our perspective intervention (i.e., a “passive” intervention that did not ask participants to take control), but further research would be needed to make this determination. Also, given that our interventions were inspired by emotion regulation and reappraisal [78, 79, 82, 143], it is possible that excluded individuals are better able to control and adapt their emotions following our interventions, thus allowing them to perform better in the team-reward task. In sum, while it is beyond the scope of the current paper to adjudicate between these numbness and emotion-based frameworks, our main results (and even some of our more nuanced findings related to the effectiveness of our interventions) appear consistent with the emotion-based account of social exclusion.

The third and final stage relates to *consequences*, or the behavioral outcomes that arise after the first two stages of exclusion. Once someone has been excluded, they can have either a *prosocial* response (thoughts and behaviors that have positive social consequences and/or aim to improve interpersonal connections) or an *antisocial* response (thoughts and behaviors that have negative social consequences and/or amplify the division between the group and excluded individual). Several meta-analyses and theoretical papers have highlighted patterns and boundary conditions for when and where these divergent responses occur [8, 9, 11, 16, 20–22], but our results fall squarely in the antisocial category: Excluded participants not only worked less hard for their teams, but they did so at the expense of their own earnings and by selectively punishing teammates from the excluding group. This is consistent with previous reports of aggressive or vengeful acts following exclusion [32–39], even when such behaviors are risky or self-defeating [30, 40–43]. At work, these antisocial responses can lead to declines in employee performance [21, 49–51], citizenship [52], satisfaction [20], and well-being [53]. This is especially concerning because antisocial responses also tend to add to the separation between the individual and excluding group, creating a pernicious and ever-growing cycle of ostracism in the workplace (e.g., organizational shunning; [42, 144]).

But why did we find such consistent evidence for antisocial responses? One possibility has to do with the reward task: Our reward trials were simple and straightforward (repeatedly adding numbers together), but if participants instead had the chance to “redeem themselves” with a more meaningful task (e.g., devising creative solutions to a difficult problem), we may have seen excluded participants actually work *more* for their teams (in order to regain meaning and self-esteem; [9, 11, 21]). Nevertheless, another plausible interpretation of our findings is the most straightforward one: Exclusion threatened participants’ basic psychological needs for control and belonging [9, 21], so in order to replenish and rebalance those needs [47, 48], those participants engaged in vengeful behaviors towards their excluders [32, 60, 61]. Since aggression is often regarded as an act of control [145], engaging in antisocial behaviors may be an easier route to regain that desired control and recognition, compared to other prosocial behaviors [11]. Of course, this will also depend on other contextual factors surrounding the excluding event (and any subsequent perceptions of unfairness). The literature on revenge and retributive justice has shown that the nature of antisocial responses hinges on social norms and values (e.g., intentionality and group structure; [62, 64, 65]), which can lead to workplace sabotage [66, 67]. While these questions were not central to our main hypotheses in this paper, future research might examine this mechanistic account of control and its boundary conditions.

The consistent evidence we observed for antisocial reactions to exclusion parallels other findings on the negative effects of workplace exclusion, including substantial reductions in employee engagement and productivity [6, 44, 146], along with considerable losses in revenue [147, 148]. Nevertheless, while our research was an important step, our paradigm was a highly controlled experiment, and as such, there are many additional factors that bear on exclusion in professional settings. For example, the negative effects of exclusion are influenced by organizational antecedents like racial or ethnic diversity [149], the social costs or expectations associated with ostracizing others [15, 150, 151], the frequency with which exclusion occurs in the company [152], the level of effort and task interdependence [153, 154], and chronic stress levels at work [155]. In addition, gender-specific antisocial responses to ostracism have been reported [156], although notably we observed no reliable gender-specific behavioral patterns in our own findings. Future research might use our findings as a starting point to develop more contextualized workplace interventions. Finally, we think our findings may be especially applicable for virtual teams, which represent an increasingly common setup among modern corporate environments [157]. Indeed, virtual teams present unique challenges to workers and managers in producing effective outcomes [158, 159], in part because of added difficulties in communication and establishing trust, issues with making consistent task progress, and problems with facilitating consistent learning among team members [160]. It would be interesting to examine if employees working in virtual teams are especially susceptible to feelings of social exclusion, whether such feelings moderate the observed performance difficulties associated with virtual teams, and whether interventions based on our ideas might serve as useful mitigation strategies. Taken together, these results suggest the applicability of our research to teams and organizations, but further research is needed on real-world teams, as well as moderators at the organizational level.

Ostracism is a scourge [1], especially in the workplace [3, 6], with serious ramifications for employee well-being and company performance [2]. Our findings provide new empirical evidence for the negative effects of social exclusion on team-based outcomes and offer a set of applied interventions to mitigate these effects. Our interventions were successful; in all cases, they increased the willingness of excluded individuals to work on behalf of their teams, and in some cases, they even eliminated the negative effects of exclusion altogether. We invite future applied research on social ostracism to add to our promising portfolio of practical interventions. In doing so, we can begin to make meaningful inroads towards a more inclusive and productive workplace.

## Supporting information

S1 File (DOCX)
