# Supplementary material for: Outcasts and saboteurs: Intervention strategies to reduce the negative effects of social exclusion on team outcomes
Source: PLoS One. 2021 May 6;16(5):e0249851. doi: 10.1371/journal.pone.0249851 (PMC8101916; doi:10.1371/journal.pone.0249851)
Supplement: S1 File — (DOCX) [file pone.0249851.s001.docx]

**Study 4: Analysis and Results for Inclusion Participants (*n* = 290)**

**Manipulation Check**

Similar to the main (exclusion) participants, we did not expect any differences by intervention condition for inclusion participants in Study 4 on our manipulation check measures. This prediction was confirmed, and the results are displayed in Fig S1. We did not observe any differences by intervention group on feeling ignored (*F*(3, 286) = 0.91, *p* = .44, 𝜂^2^ = .009), feeling excluded (*F*(3, 286) = 1.83, *p* = .14, 𝜂^2^ = .019), feelings of belonging (*F*(3, 286) = 0.89, *p* = .45, 𝜂^2^ = .009), or estimates of ball toss percentage (*F*(3, 286) = 0.86, *p* = .46, 𝜂^2^ = .009).


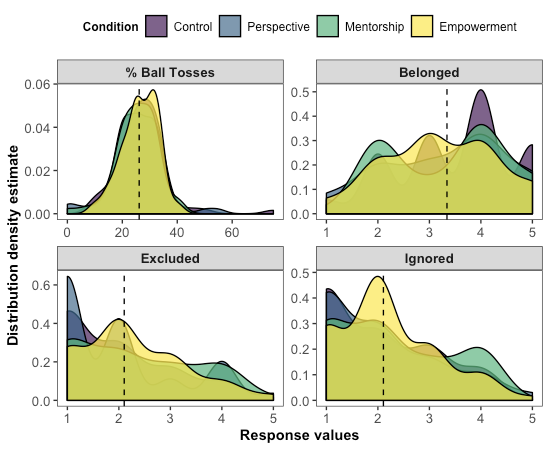


**Fig S1.** **Distributions of manipulation check measures by intervention condition for *included participants* in Study 4.** Dotted lines show the overall mean response value for each measure. There were no differences by intervention group for any of the measures. For estimating the percentage of ball tosses, participants typed a number between 0 and 100. For the questions on feelings of belonging, being excluded, and being ignored, participants answered using 5-point scales (1 = *Not at all*; 5 = *Extremely*).

**Reward Task**

Fig S2 displays the main results from the team reward task for *included participants* in Study 4. A main effect of Intervention (𝜒^2^(3) = 31.1, *p* < .001) revealed that participants in the perspective condition completed the fewest number of reward tasks (*M_perspective_* = 18.13, *SD_perspective_* = 13.61), which was significantly different from participants in the control condition (*M_control_* = 20.16, *SD_control_* = 17.81; *z* = 3.15, *p* = .007), mentorship condition (*M_mentorship_* = 21.17, *SD_mentorship_* = 20.03; *z* = 4.50, *p* < .001), and empowerment condition (*M_empowerment_* = 21.45, *SD_empowerment_* = 19.34; *z* = 5.05, *p* < .001). There were no differences among the control, mentorship, or empowerment conditions (*z*s ≤ 1.92, *p*s ≥ .16).


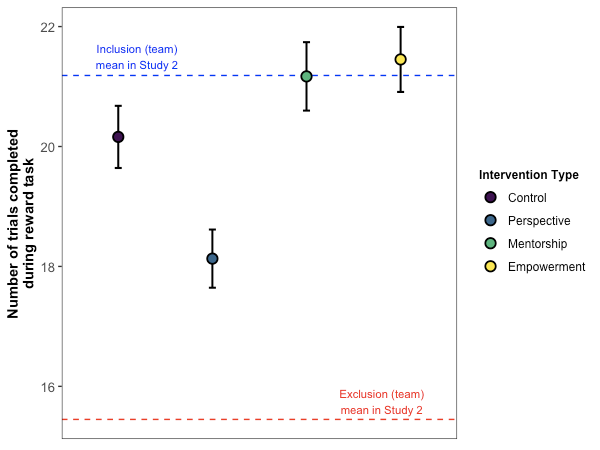


**Fig S2. Main results from the team reward task for *included participants* in Study 4.** The number of completed reward tasks is shown on the *y*-axis, and intervention conditions are color-coded. The blue dotted line shows the grand mean reward task completion for all *inclusion* participants in Study 2 that were earning rewards on behalf of their *teams*. The red dotted line shows the grand mean reward task completion for all *exclusion* participants in Study 2 that were earning rewards on behalf of their *teams*. Grand means for Study 2 inclusion and exclusion participants are shown as a reference, given the similarity in task design and reward structure. Error bars show ±1 SE.
